# Altered glycosylation of several metastasis-associated glycoproteins with terminal GalNAc defines the highly invasive cancer cell phenotype

**DOI:** 10.18632/oncotarget.28167

**Published:** 2022-01-10

**Authors:** Elham Khosrowabadi, Tomasz Wenta, Salla Keskitalo, Aki Manninen, Sakari Kellokumpu

**Affiliations:** ^1^University of Oulu, Faculty of Biochemistry and Molecular Medicine, Oulu, Finland; ^2^Institute of Biotechnology, University of Helsinki, Helsinki, Finland

**Keywords:** cancer, invasion, glycosylation, glycocode, terminal GalNAc, Helix Pomatia agglutinin (HPA)

## Abstract

Several distinct metastasis-associated glycosylation changes have been shown to promote cancer cell invasion and metastasis, the main cause of death of cancer patients. However, it is unclear whether their presence reflects cell- or tissue-specific variations for metastasis, or species needed to drive different phases of the metastatic cascade. To address this issue from a different perspective, we investigated here whether different cancer cell lines share any glycotopes that are common and important for their invasive phenotype. By using lectin microarray glycan profiling and an established myoma tissue-based 3D invasion assay, we identified a single glycotope recognized by *Helix Pomatia* agglutinin (HPA), whose expression level in different cancer cells correlated significantly with their invasive potential. Lectin pull-down assay and LC-MS/MS analysis in highly- (A431 and SW-48) and poorly invasive (HepG2 and RCC4) cancer cells revealed ~85 glycoproteins of which several metastasis-promoting members of the integrin family of cell adhesion receptors, the epidermal growth factor receptor (EGFR) and the matrix metalloproteinase-14 (MMP-14) were among the abundant ones. Moreover, we showed that the level of the GalNAc glycotope in MMP-14, EGFR, αV-, β1- and β4 integrin in highly and poorly invasive cancer cells correlated positively with their invasive potential. Collectively, our findings suggest that altered glycosylation of several metastasis-associated glycoproteins with terminal GalNAc drives the highly invasive cancer cell phenotype.

## INTRODUCTION

Glycans are among the fundamental building blocks of life and play a key role in the development and physiology or pathology of multicellular organisms via mediating various cell-cell, cell-extracellular matrix, or cell-pathogen interactions [1–6]. In accord with this, several developmental disorders that are associated with impaired biosynthesis of glycans, have been identified in humans [7]. In all eukaryotes, the Golgi apparatus is the main site of glycosylation of various cell surface proteins and lipids that together form the glycocalyx, the sugar “coat” on the plasma membrane of all eukaryotic cells [8–9]. In the Golgi, and other organelles (ER and the plasma membrane) where some glycans are also made, their biosynthesis is driven by special enzymes called glycosyltransferases that add specific sugar residues to growing glycan chains in a precise order and linkage type despite of not using any template for synthesis. Because of this, cell surface glycans do not represent random polymers of sugars but rather, a dynamic set of distinct glycans that can be cell-, protein- or lipid-specific [9, 10]. In part, this is due to transcriptional programs that regulate the levels of glycosylation enzymes in the Golgi [11–13] and in part to environmental factors such as luminal pH and redox state that are needed to maintain Golgi homeostasis and co-operative functioning of the glycosyltransferases [14, 15].

During the last 3 decades, it has become clear that the Golgi apparatus in cancer cells is both structurally and functionally impaired. It typically has a fragmented morphology and produces shorter, more branched, and differentially fucosylated, sialylated, or sulphated glycans than those produced by non-malignant cells [16–20]. Together with genetic and epigenetic changes, altered glycans have been shown to contribute to cell growth and survival, development of tumors, and their metastatic spread via modulating cell adhesion, migration, invasion, apoptosis, immune evasion, and resistance to chemotherapy [17, 21–30]. Previously, several distinct metastasis-promoting glycotopes have been identified separately by different groups [28, 31–40]. These include increased core fucosylation of N-glycans, increased expression of O-linked GalNAc (Tn) or the sialyl-Lewis A and X (SLea, SLex) antigens, and increased branching of N-glycans with the β-(1,6)-linked GlcNAc. They all seem to have prognostic utility in detecting and treating aggressive cancers, given their prevalence in various cancers and predominant localization at the plasma membrane where they are easily accessible to potential therapeutic drugs.

However, the mechanistic details on how these altered glycotopes enhance metastasis are less clear. Moreover, the fact that there are several distinct metastasis-promoting glycotopes is puzzling. One possibility is that each one has a special purpose during metastasis, be it cell dissemination, migration, degradation of the extracellular matrix (ECM), intra- and extravasation, or colonization at adjacent or remote sites. Alternatively, their presence may reflect cell-, tissue- or cancer type-specific glycoforms to enhance the metastatic potential in different surroundings. Unravelling which one is the case is important but not trivial, given that glycosylation can be both protein- and site-specific. Also, the methodology that is needed to faithfully mimic metastasis *in vivo* and at the same time allow dissection of different metastatic phases from each other, is not in routine use.

In this study, we decided to take a different approach and investigate whether different cancer cell types share any common glycotopes that are important for their invasive potential. By using an established myoma tissue-based 3D invasion assay [41], lectin microarray glycan profiling, correlation, and multiple linear regression analyses we identified a single GalNAc glycotope that is recognized specifically by the *Helix Pomatia* agglutinin (HPA) and is important for the highly invasive cancer cell phenotype. Moreover, lectin pulldown and LC-MS/MS analyses in highly and poorly invasive cell lines also revealed several distinct and abundant metastasis-promoting glycoproteins that display increased HPA binding in highly invasive cells compared to poorly invasive cells. These findings suggest that altered glycosylation of these metastasis-promoting glycoproteins with a terminal GalNAc is the key to the highly invasive cancer cell phenotype.

## RESULTS

### Cancer cell lines display variable invasive potential in a 3D invasion assay

The geno- and phenotypic characteristics of the nine different cancer cell lines used in this study are depicted in Table 1. Overall, the cells display variable karyotypes and have several different tissue origins. Four of the cell lines are derived from colon adenocarcinomas (SW48, DLD-1, CaCo-2, and HT-29), two from breast cancer metastases (MCF-7, MDA-MB231), and the rest three (A431, RCC4, and HepG2) represent skin, kidney, and liver carcinomas. Except for HepG2, they all form tumors in nude mice. In certain cases, non-malignant COS-7 cells from the kidney of African green monkey were used for comparison.

**Table 1:** Cellular characteristics of the different cancer cell lines

Cell Type	Gender	Age	Tissue of origin	Cancer type	Cell type	Karyotype (Chr. Number)
SW-48	Female	82 years	colon	adenocarcinoma	epithelial	Diploid 47 (+7)
DLD-1	Male	adult	colon	adenocarcinoma	epithelial	Pseudodiploid (46)
CaCo-2	Male	72 years	colon	adenocarcinoma	epithelial	Tetraploid (96)
HT-29	Female	44 years	colon	adenocarcinoma	epithelial	71
A431	Female	86 years	skin	carcinoma	epithelial	Triploid (74)
HepG2	Male	15 years	liver	carcinoma	epithelial	55
RCC4	?	?	kidney	carcinoma	epithelial	? (VHL^−/−^)
MCF-7	Female	69 years	breast (metastasis)	adenocarcinoma	epithelial	82
MDA-MB231	Female	51 years	breast (metastasis)	adenocarcinoma	epithelial	64

**Figure 1:**
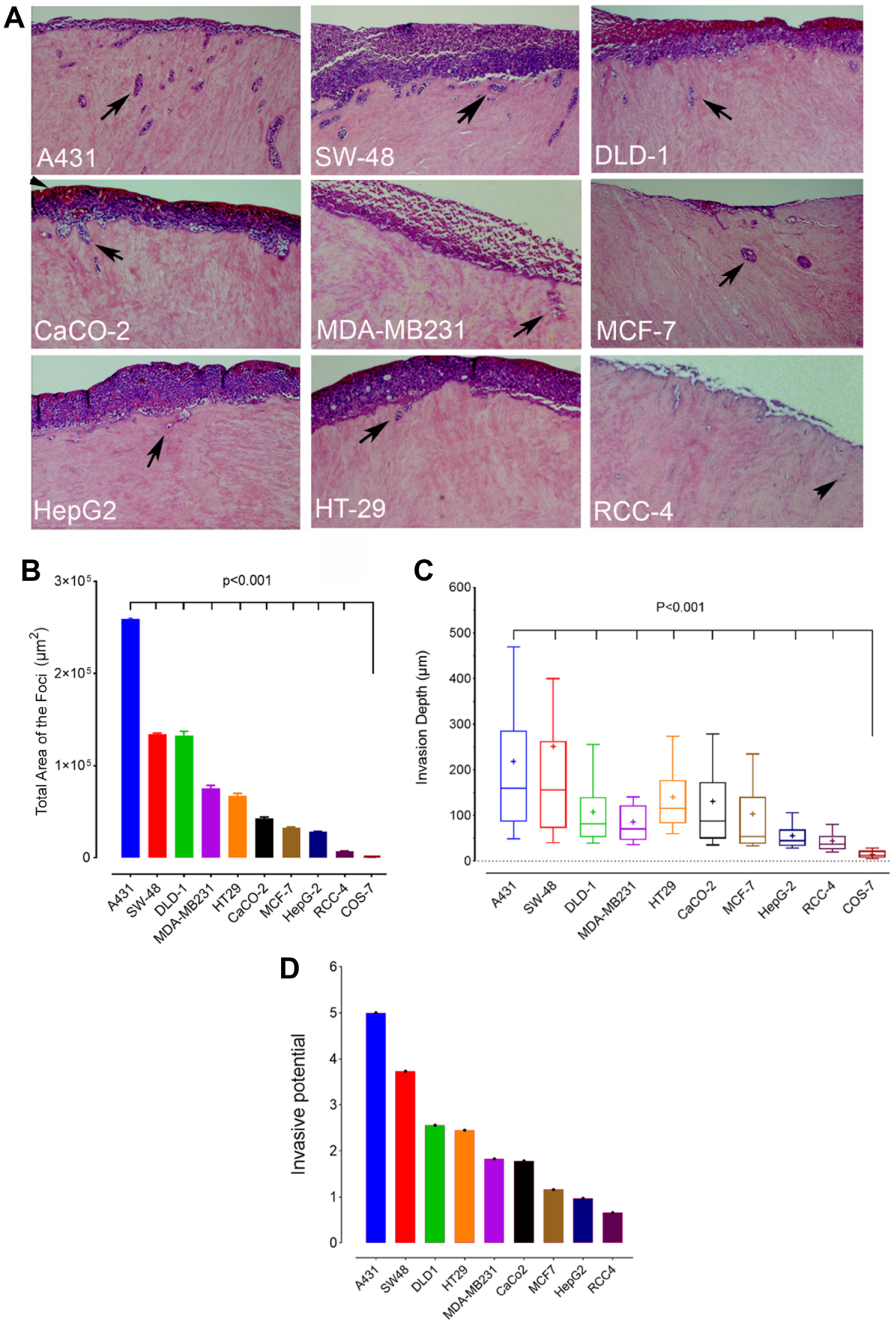
Invasion potential of different cancer cell lines. (**A**) Myoma-tissue-based 3D invasion assay. Cells were seeded on top of myoma discs, allowed to grow for 21 days before fixing and processing for histochemical staining. Sections were cut perpendicularly to the seeded cell layer, stained, and imaged before quantification. Representative figures of the invasive foci inside the myoma tissue are shown (arrowheads). (**B**) Total invasion area (μm^2^) of the foci in each cell line. (**C**) The median invasion depth of the foci from the seeded cell layer. Twelve sections from each myoma disc (*n* = 2/24) were used for the quantification with ImageJ software. The whiskers indicate 10th to 90th percentiles. (**D**) A bar graph showing the relative invasive potential of each cancer cell type. The values were calculated by scaling the medians of the total area and the median depth using scores from 5 (high) to 0 (low). Invasive potential was calculated as the mean of the two scores.

To determine first the invasive potential of the different cancer cell lines, we used an established 3D human myoma tissue-based invasion assay that mimics well the *in vivo*-conditions in that cells need to degrade the myoma tissue before being able to invade into the tissue. In brief, cells were seeded on top of the myoma slices, allowed to grow for 3 weeks before processing for histochemical staining and quantification of the invasive foci present in each section (Figure 1A, arrows). Because some cancer cell lines displayed few and large foci deep inside the tissue while in others the foci were small and numerous and near the seeded top cell layer, it was necessary to quantify both the total area (Figure 1B) and the median depth (Figure 1C) of the foci. To get a reliable estimate of the invasion potential, the total area and median depth were scored mathematically using a scale from five (high) to zero (low). The invasive potential (index) was then calculated as the mean of the two (Figure 1D). Based on scoring, skin A431 and colon SW-48 cells displayed the highest invasive potential while liver HepG2 and kidney RCC4 cells had the lowest invasive potential of all the cell types studied. A431 cells were roughly 7.5-fold more invasive than the least invasive RCC4 cells. No invasive foci were detected in COS-7 cells, consistent with their non-malignant phenotype (Figure 1B and 1C).

### Glycosylation differences between cancer cell lines are both tissue- and cell type-dependent

**Figure 2:**
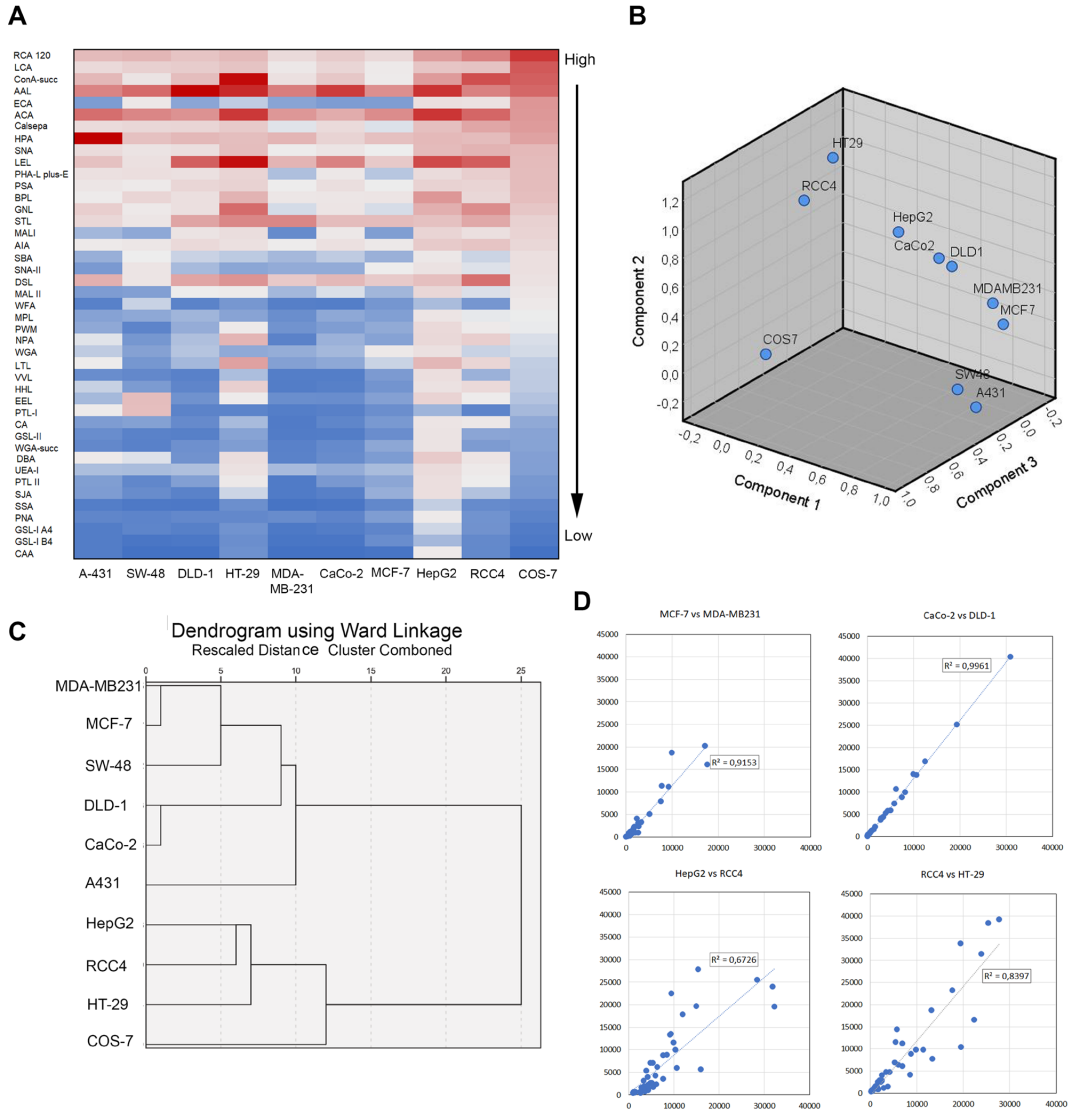
Comparison of glycosylation profiles between different cancer cell types. (**A**) Heat map representation of the lectin microarray glycan profiles. The rows represent normalized median intensity values of each lectin. Three independent samples were used for determination of the intensity values in each cell line. The color scale from blue to red indicates low to high signal intensities. Non-malignant COS-7 cells were used as a reference cell line for ranking. (**B**) Comparison of the glycan profiles by using principal component analysis tool in Excel. Normalized intensity values for each lectin were used for the analysis. The number of components is determined automatically from the input values. (**C**) Hierarchical clustering analysis of the glycosylation differences or similarities between different cancers cell type. Normalized median intensity values of each lectin, the SPSS software with Ward linkage analysis and Euclidean correlation coefficient as the distance metric were used for the analysis. (**D**) Comparison of the glycosylation profiles between subcluster forming cell lines. In each plot, normalized median intensity values were plotted against each other. The lines represent linear correlation equations. The R2 values are shown for each plot.

Next, we determined glycosylation profiles of the nine cancer cell lines by using lectin microarray glycan profiling. To allow direct comparisons between the cancer cell lines, the calculated medians from three independent samples (36 measurements points) were normalized against α-tubulin before further analyses. Overall, heat map analysis (Figure 2A) showed that with few exceptions, the same lectins and their specific glycotopes were amongst the most or the least abundant irrespective of the cancer cell line in question, when COS-7 cells were used as a reference cell line. However, principal component analysis (PCA) with SPSS showed marked differences in glycan signatures between the different cancer cell lines, and between non-malignant COS-7 cells and the cancer cell lines (Figure 2B). Interestingly, PCA analysis identified three distinct cell pairs formed by A431 and SW-48 cells, MCF-7 and MDA-MB231 cells, and CaCo-2 and DLD-1 cells that were more closely related to each other than the other cell lines used in the study. In further support, hierarchical clustering with Ward linkage analysis together with Euclidean correlation coefficient as the distance metric showed that the glycosylation profiles (Supplementary Figure 1) of the two cell pairs (MCF-7 and MDA-MB23; CaCo-2 and DLD-1) were the closest homologs in terms of their glycan signatures (Figure 2C) while A431 and SW-48 cells were more distant and formed separate branches in one of the main subclusters. The other main subcluster was formed by the three poorly invasive cell lines: HepG2 (liver), HT-29 (colon), and RCC4 (kidney). Non-invasive COS-7 cells were also classified to this second main subcluster, suggesting their closer relationship with these three cancer cell types than with the other subcluster forming cell lines. Correlation and regression plots between the identified cell pairs confirmed their similar glycosylation patterns in each case (Figure 2D). Given their different tissue origins, it is likely that the variable glycosylation signatures reflect both tissue- (MCF-7 and MDA-MB231) or cell type-specific (DLD-1 and CaCo-2) glycosylation differences.

**Figure 3:**
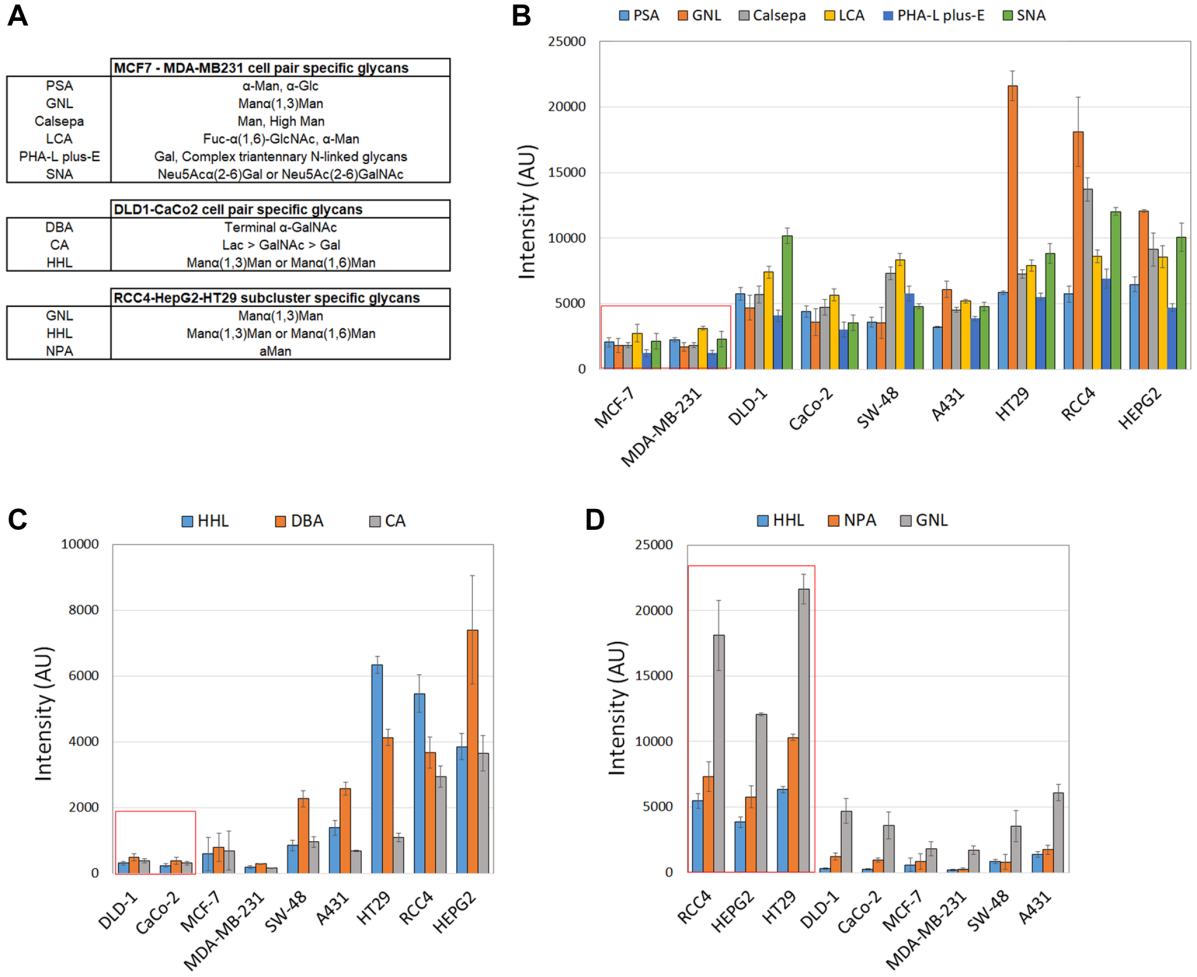
Identification of lectins that are specific for each clustered cell pair. Lectins that define clustered cell pairs were identified by using conditional formatting algorithms embedded in Excel. In brief, similar lectin binding intensities were searched for each clustered cell by setting an acceptable intensity limit to 1.5 × SD. Any values within these limits were designated as similar whereas the ones exceeding these limits were designated as dissimilar. Specific lectins for each cell pair were then selected based on its similarity between the cell pair and dissimilarity in the other cell lines. Normalized median intensity values (±SD) were used for comparisons. (**A**) Selected lectins and their sugar specificities specific for each clustered cell pair. (**B**) Histogram showing lectin binding intensities specific for the clustered MCF-7/MDA-MB231 cell pair (marked by red box). (**C**) Histogram showing lectin binding intensities specific for the clustered DLD-1/CaCo-2 cell pair. (**D**) Histogram showing lectin binding intensities specific for the clustered RCC4, HepG2 and HT-29 cell lines.

Conditional formatting algorithms embedded in Excel were used next to identify glycotopes that are specific for each cell pair. To accomplish this, normalized lectin binding intensities (Figure 3) in each cell pair were classified and determined to be either similar or dissimilar depending on the cell line. For example, by using MCF-7 cells as a reference, we sought lectins whose binding intensities were similar with MDA-MB231 cells but dissimilar in the other cell lines. This approach yielded 6 lectins (PSA, GNL, Calsepa, LCA, PHA-L/E, and SNA) that specifically separate MCF-7 and MDA-MB231 cell pair from the other cell lines studied (Figure 3A and 3B). These lectins recognize various N-glycosylation intermediates, suggesting that MCF-7 and MDA-MB231 cells differ from the other cell lines mostly by their altered N-glycosylation status. The two colorectal cell lines (DLD-1 and CaCo-2) in turn displayed low binding to DBA, CA and HHL lectins (Figure 3A and 3C), suggesting that low levels of certain GalNAc- and mannose-containing glycotopes are typical for this cell pair. In contrast, poorly invasive RCC4 and HepG2 cells as well as moderately invasive HT-29 cells displayed high binding to HHL, GNL and NPA lectins (Figure 3A and 3D). These lectins are specific for various mannose-containing glycotopes and suggest that their high levels in the three cells lines can distinguish them from the other cancer cell types.

To visualize the main glycosylation changes betweenRCC4 cell and the other cancer cell lines, we subtracted normalized lectin binding intensities of RCC4 cells from those of the other cell lines to get so called subtracted fingerprints. Statistically significant fingerprints are shown in Supplementary Figure 2. The main differences included increased levels of truncated O-glycans, decreased levels of mannose-containing N-glycans, increased levels of specifically core-fucosylated N-glycans, and decreased levels of terminally glycosylated N-glycans. These changes, therefore, demonstrate that cancer cells differ markedly in their O- and N-glycan signatures.

### Cancer cell invasive potential correlates with *Helix Pomatia* lectin (HPA) binding

**Figure 4:**
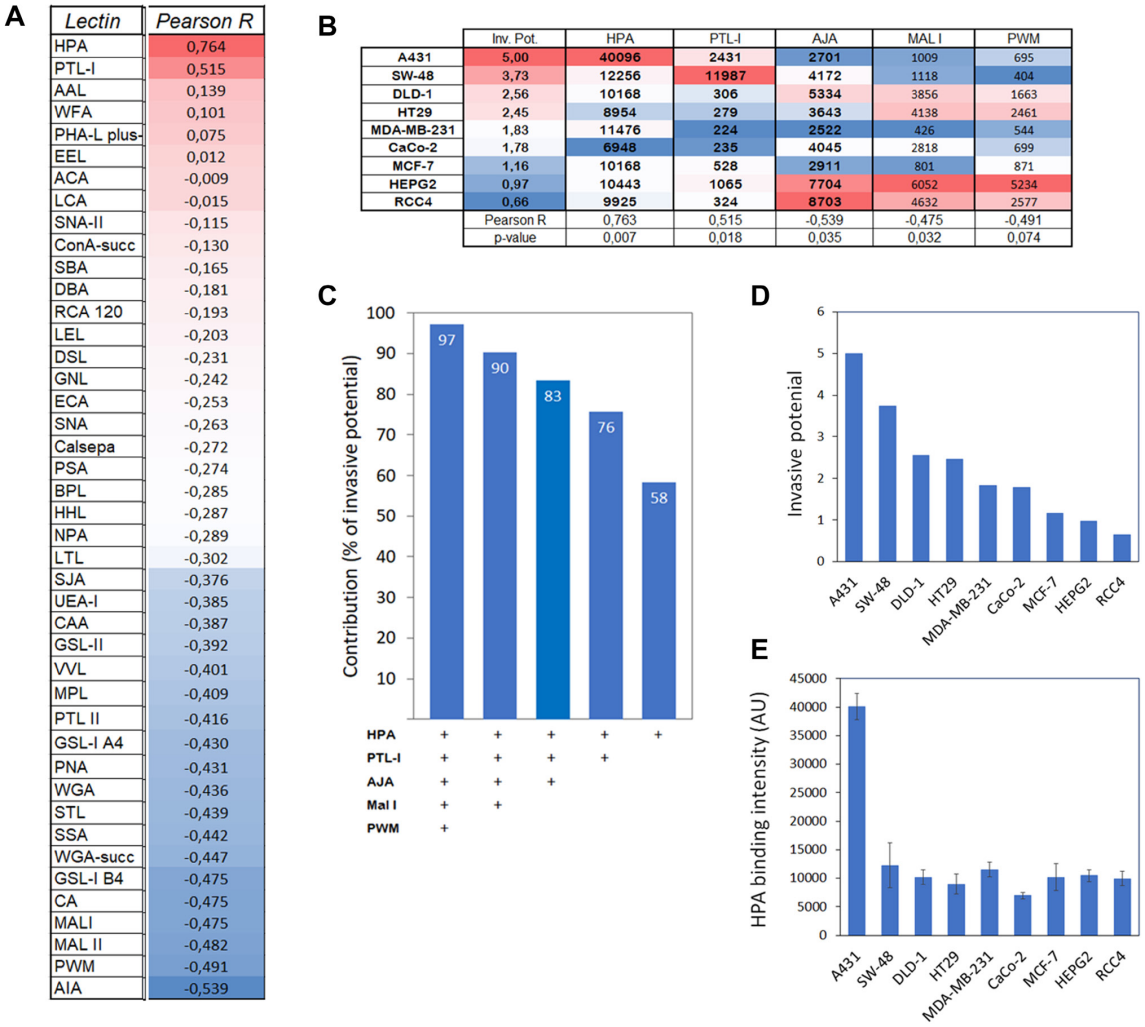
Correlation and impact of HPA binding to cancer cell invasive potential. (**A**) Correlation analysis between lectin binding and invasive potential of different cancer cell types. Normalize median intensity values for each lectin and relative invasive potential (Figure 1D) were used for the analysis with Excel’s data analysis tool pack. Pearson R values are shown and ranked from high to low and marked with red (high) and blue (low) colors. (**B**) Correlation analyses of selected lectins with their normalized median intensity values in relation to invasive potential of different cancer cell types. Pearson R values and statistical significance of the correlation are shown at the bottom of the table. Regression analysis with ANOVA was used for testing the statistical significance. (**C**) Multiple linear regression analysis showing the contribution of each lectin to cancer cell invasive potential. Each bar represents the contribution (as percentages) of the lectins used for the analysis (bottom). (**D** and **E**) Comparison of the cancer cell invasive potential (D) with the level of HPA binding glycotopes (E) in different cancer cell lines. Correlation and regression analyses showed a significant correlation between these two variables (R-value of 0.76 and *p*-value of 0.007^**^).

To find out next whether cancer cells possess any common glycotopes that are important for their invasive phenotype, we performed correlation and multiple linear regression analyses using algorithms embedded in Excel data analysis tool pack. Out of the 43 lectins in the array (Figure 4A), only five lectins (HPA, PTL-1, AJA, MAL I, PWM) were found to correlate either positively or negatively with the invasive potential of the cancer cells (Figure 4B). Multiple linear regression analyses further demonstrated that these five lectins accounted for 97% of the variation observed in the cancer cell invasive phenotype (Figure 4C). By omitting the least significant contributor from the list after each subsequent round, it was found that HPA (specific for GalNAc glycotope) alone accounted for 58% of the variation (Figure 4C). Its levels in different cancer cell lines also correlated positively with cancer cell invasive potential (R = 0.763: *p* < 0.007; Figure 4B, 4D and 4E). Together with PTL-1, HPA accounted for 76% of the variation, while the rest (AJA, MAL I, PWM) correlated negatively and accounted for 7% each. Thus, increased expression of HPA-binding glycotopes (O-linked GalNAc) in cancer cells appears to be the main factor promoting cancer cell invasive phenotype. Yet, decreased expression of AJA, PWM and MAL 1 specific glycotopes (O-linked Galβ(1,3)GalNAc, N-glycan branching and α-2,3-sialylation, respectively) also contribute to some extent.

### Identification of HPA binding glycoproteins by lectin blotting and mass spectrometry

**Figure 5:**
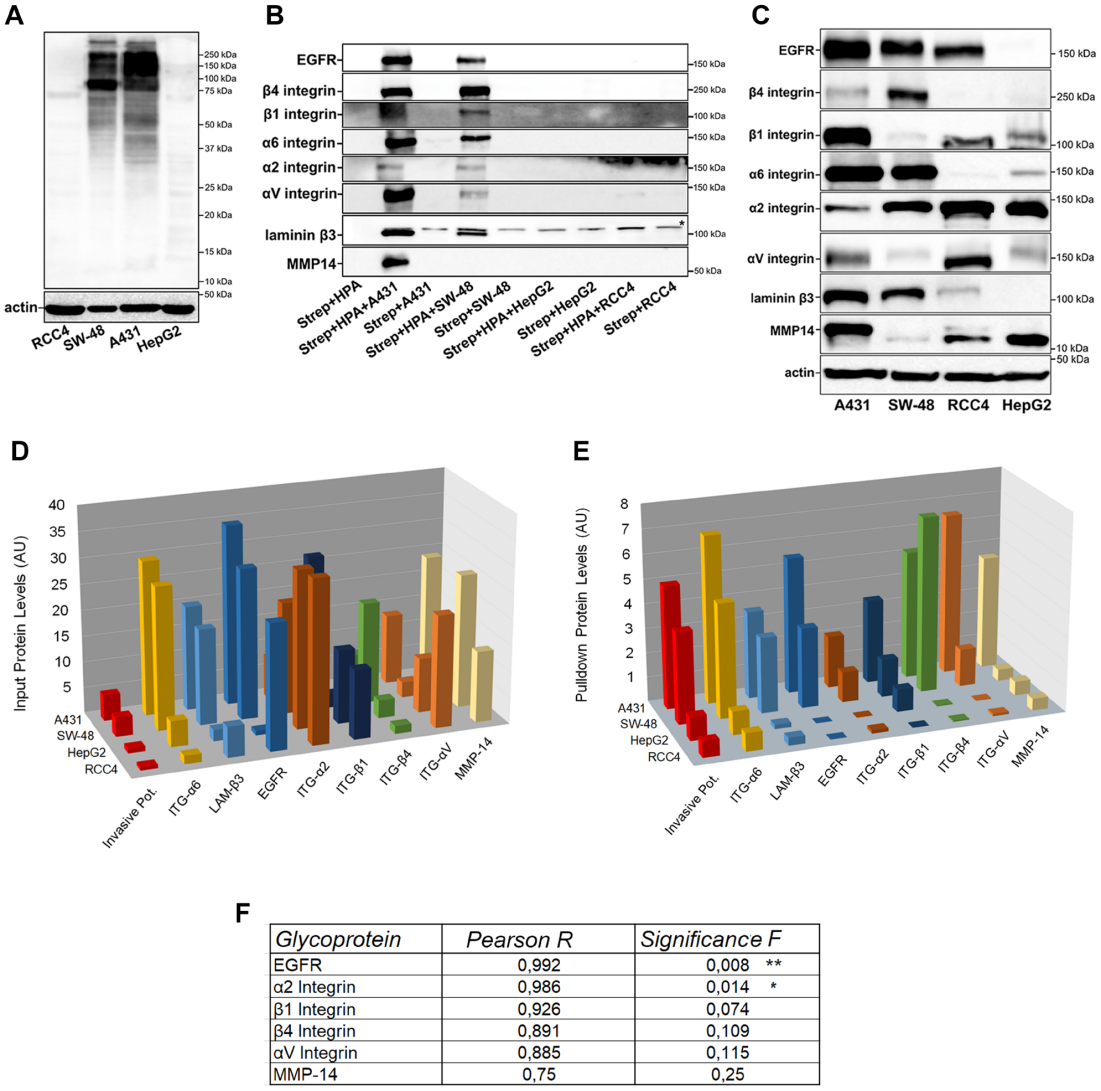
Identification of HPA binding glycoproteins and their comparison between highly and poorly invasive cancer cell types. (**A**) HPA-binding proteins in highly (A431 and SW-48) and poorly (RCC4 and HepG2) invasive cancer cell samples as revealed by lectin blotting with biotinylated HPA. Complexes were visualized using HRP-conjugated streptavidin. A representative blot is shown. (**B**) Immunoblotting of selected HPA-binding glycoproteins after lectin pull-down with relevant antibodies in highly (A431, SW-48) and poorly (RCC4 and HepG2) invasive cancer cells. The star (^*^) in laminin β3 blot denotes a non-specific band. A representative blot is shown. (**C**) Immunoblotting of the HPA binding glycoproteins in input samples of the same cell types. A representative blot is shown. (**D**) Quantification of HPA pull-down protein levels in the immunoblot (B). ImageJ software was used for quantification of the band intensities as arbitrary units (AU). (**E**) Quantification of protein input levels in the immunoblot (C). ImageJ software was used for quantification of the band intensities as arbitrary units (AU). (**F**) Correlation analysis between HPA pull-down protein levels with invasion potential. Excel data analysis tool pack’s correlation and regression analyses with ANOVA were used to get the Pearson R and significance values. The stars (^*^) denote the statistical significance of *p* < 0.01^**^ and *p* < 0.05^*^, respectively.

Since HPA lectin has been previously suggested to have prognostic utility in detecting metastatic breast and colorectal cancer cell lines [42–47], we decided to identify glycoproteins that carry the glycotope specific for the HPA lectin using highly - (A431 and SW-48) and poorly invasive (RCC4 and HepG2) cells as our targets. It was also anticipated that such identification would also give new insights into mechanistic details for why this glycotope enhances cancer cell invasive potential. To accomplish this, we first used lectin blotting with HPA to visualize HPA binding proteins and their levels in different cell lysates. HPA blotting (Figure 5A) revealed several prominent bands with a MW of ~400–600 kDa, ~240 kDa, ~160 kDa, ~130 kDa, ~80 kDa, 50 kDa, and 45 kDa) in highly invasive A431 and SW-48 cells. Importantly, the bands were almost undetectable in poorly invasive HepG2 and RCC4 cells.

Next, HPA-binding proteins were pulled down with the lectin before their identification by liquid chromatography and tandem mass spectrometry (LC-MS/MS). Altogether, we identified ~85 glycoproteins that potentially bind HPA (Supplementary Table 1). After classifying the proteins by their abundance (peak heights) in highly invasive A431 cells, we selected the 60 most abundant ones and tested how well their levels correlate with the invasive potential of these same cells. Using a Pearson R cutoff of >0.77, we were left with 35 glycoproteins of which more than half represented glycoproteins with a known role in cell adhesion, migration, cell signaling, or metastasis (Table 2). Of these, we selected eight glycoprotein candidates (epidermal growth factor receptor (EGFR), matrix metalloproteinase-14 (MMP-14), β4-, β1-, α6-, α2- and αV-integrin and laminin β3 for further analyses. Quantification of the bands after western blotting showed first that all these proteins were expressed at higher levels in highly invasive A431 and SW-48 cells than in poorly invasive HepG2 and RCC4 cells, as expected (Figure 5B, 5D). However, when compared to protein input levels (Figure 5C, 5E), we noticed that out of these eight glycoproteins, α6 integrin and laminin β3 levels in pull-down and input samples matched well with each other, and in fact, did show a significant correlation with each other (Supplementary Figure 3A). Similar results were also obtained by calculating their ratios (Supplementary Figure 3B). These data suggested that these two proteins have the same amount of GalNAc glycotope in highly and poorly invasive cancer cells.

**Table 2:** The list of invasion-promoting glycoprotein candidates identified by LS-MS/MS from HPA pull-down samples

Gene	Glycans	ID (Uniprot)	Protein name	Function (based on Uniprot ID)	Pearson R
***ITGB4***	**N+O**	**P16144**	**Integrin beta-4**	**Cell adhesion, migration, motility, signaling**	**0,997**
***ITGA6***	**N+O**	**P23229**	**Integrin alpha-6**	**Cell adhesion, migration, motility, signaling**	**0,997**
*ASPH*	N+O	Q12797	Aspartyl/asparaginyl beta-hydroxylase	Hydroxylation of Asp or Asn (EGF-like domains)	0,995
*GOLM1*	N+O	Q8NBJ4	Golgi membrane protein 1	Regulation of lipid/protein metabolism	0,992
*HSP90AB1*	N+O	P08238	Heat shock protein HSP 90-beta	Protein folding	0,989
*TFRC*	N+O	P02786	Transferrin receptor protein 1	Iron transport and homeostasis	0,971
***BSG***	**N+O**	**P35613**	**Basigin**	**Cell adhesion, migration, motility, signaling**	**0,964**
*ST14*	O	Q9Y5Y6	Suppressor of tumorigenicity 14 protein	Degradation of the ECM	0,959
*ATP5F1B*	O	P06576	ATP synthase subunit beta, mitochondrial	ATP synthesis	0,957
*PTGFRN*	N+O	Q9P2B2	Prostaglandin F2 receptor negative regulator	Regulates Prostaglandin receptor activity	0,956
***EGFR***	**N+O**	**P00533**	**Epidermal growth factor receptor**	**Migration, signaling, malignant transformation**	**0,945**
*SPINT1*	N+O	O43278	Kunitz-type protease inhibitor 1	Endopeptidase inhibitor	0,934
*PRKDC*	N+O	P78527	DNA-dependent protein kinase catalytic subunit	DNA damage sensor	0,924
***ITGA2***	**N+O**	**P17301**	**Integrin alpha-2**	**Cell adhesion**	**0,924**
***PLXNB2***	**N+O**	**O15031**	**Plexin-B2**	**Invasive growth, migration, signaling**	0,921
***DSG2***	**N+O**	**Q14126**	**Desmoglein-2**	**Cell adhesion**	**0,918**
***F11R***	**N+O**	**Q9Y624**	**Junctional adhesion molecule A**	**Cell adhesion, tight junction formation, migration**	**0,915**
***PLXNA1***	**N+O**	**Q9UIW2**	**Plexin-A1**	**Cell adhesion (homotypic)**	**0,890**
***EPCAM***	**N+O**	**P16422**	**Epithelial cell adhesion molecule**	**Cell adhesion**	**0,890**
***ITGB1***	**N+O**	**P05556**	**Integrin beta-1**	**Cell adhesion**	**0,885**
*LRP1*	N+O	Q07954	Pro-low density lipoprotein receptor-related protein 1	Endocytosis	0,877
***GLG1***	**N+O**	**Q92896**	**Golgi apparatus protein 1**	**Cell adhesion**	**0,875**
*GALNT3*	N+O	Q14435	Polypeptide N-acetylgalactosaminyltransferase 3	O-Glycosylation	0,870
***LGALS3BP***	**N+O**	**Q08380**	**Galectin-3-binding protein**	**Cell adhesion**	0,847
*PSAP*	**N+O**	**P07602**	**Prosaposin**	**Lipid metabolism**	**0,838**
***LAMA3***	**N+O**	**Q16787**	**Laminin subunit alpha-3**	**Adhesion, migration, differentiation**	**0,818**
***ITGB6***	**N+O**	**P18564**	**Integrin beta-6**	**Cell adhesion**	**0,813**
***IGSF8***	**N+O**	**Q969P0**	**Immunoglobulin superfamily member 8**	**Cell motility**	**0,797**
***ITGAV***	**N+O**	**P06756**	**Integrin alpha-V**	**Cell adhesion**	**0,777**
***MMP14***	**O**	**P50281**	**Matrix metalloproteinase-14**	**Degradation of the ECM**	0,774

The proteins were classified first by their abundance and then, by their correlation (Pearson R) with cancer cell invasive potential. Bold text refers to proteins which have a known role in cell migration, adhesion, and metastasis according to the Uniprot database.

Importantly, all the other glycoproteins (EGFR, MMP-14, β4-, β1-, α2- and αV integrin) in HPA pulldown samples were markedly enriched in highly invasive cells relative to poorly invasive cells (Figure 5D), indicating that these proteins carry increased levels of a terminal GalNAc glycotope in their glycans. Of these same glycoproteins, only the EGFR and α2 integrin correlated significantly with cancer invasive potential (Figure 5F). Thus, these two proteins might be the most important for highly invasive cancer cell phenotype. However, because pulldown experiments are only semi-quantitative, the impact of the other GalNAc-carrying proteins (MMP-14, β4, β1, and αV integrins) on cancer cell invasion cannot be excluded.

## DISCUSSION

By using lectin microarray glycan profiling and a 3D myoma tissue-based invasion assay, we identified here one single glycotope, a terminal GalNAc, that is recognized by Helix Pomatia agglutinin (HPA) and that correlated significantly with the invasive potential of the nine different cancer cell types used in this study. Moreover, by using lectin pulldown and proteomics tools, we uncovered ~85 glycoproteins with either known or potential binding site for the lectin. We also identified several metastasis-associated glycoproteins including MMP-14, EGFR, αV, β1, and β4 integrins that displayed higher levels of the GalNAc glycotope in lectin pull-down samples of highly invasive cancer cell lines, in contrast to poorly invasive cells. Altogether, these findings suggest that altered glycosylation with a terminal GalNAc of these glycoproteins in highly invasive A431 and SW-48 cancer cells is the key to their high invasive potential when compared to poorly invasive HepG2 and RCC4 cancer cells.

Increased HPA binding has been previously shown to be associated with poor prognosis and development of metastases *in vivo* both in cancer patients and mouse models [42–48]. For example, Schumacher and Adam [42] showed that inoculation of HPA positive colon (HT29) or breast cancer cell lines (MCF-7, T47D) into immunodeficient mice resulted in the development of metastases in 23 out of 26 cases while metastases were non-existent or rare in HPA negative cell lines. On the other hand, previous breast cancer studies by Milde-Langosch et al. [47] suggested that HPA binding *in vivo* was associated with increased invasion. By using a Matrigel invasion assay, Rye et al. [43] in turn showed that an HPA positive melanoma cell line was more invasive than an HPA-negative control cell line. Thus, our observations are fully consistent with these findings, but they also emphasize that increased HPA binding is likely a general feature of all cancer cell types with high invasive potential. It is also of note that despite the use of different methodologies and approaches used in the above studies, the outcomes are similar.

Together, the above studies provide strong support for the view that HPA binding and the presence of a GalNAc glycotope in specified glycoproteins is an important determinant of highly invasive cancer cells. Yet, because HPA binds in addition to terminal GalNAc, a broader array of glycotopes including GalNAcα1,3Gal and GlcNAcβ1,4Gal [43, 47], the exact identity of the HPA-specific glycotope remains to be elucidated. Moreover, it is also unreasonable to expect that all lectins with a similar nominal binding specificity will give identical results, as lectin specificity can depend also on sub-terminal as well as 3D structural motifs. This phenomenon likely explains why HPA, in contrast to DBA and PTL-1, did exhibit a significant correlation with cancer cell invasive potential. In accord with this, Laferte et al. [49] have shown that glycoproteins from both colon cancer tissue and HT-29 colon cancer cells bound HPA while they displayed poor binding to DBA.

To gain insight into why HPA binding is associated with high invasive potential, it was also important to identify glycoproteins that carry the glycotope(s) for HPA. Previous work from Dwek’s Laboratory [50–52] has indicated that in metastatic tumor cells from breast and colon, HPA binding correlated with levels of α6 integrin, HnRNP family of transcription factors (heterogeneous nuclear ribonuclear proteins H1, D-like, and A2/B1), heat shock protein 27 (Hsp27), glial fibrillary acidic protein and enolase 1 (ENO1). None of these proteins were detected in the non-metastatic breast cancer cell lines. Interestingly, we also identified α6 integrin as one of the most abundant glycoproteins in highly invasive cells. Its levels together with laminin β3 in highly and poorly invasive cells also correlated with HPA binding. However, when input levels were taken into account, this correlation was lost, suggesting that the level of HPA binding sites in either glycoprotein was not different between highly and poorly invasive cells. In contrast to earlier studies, we detected several other cell adhesion receptors (including α2-, β1-, αV- and β4 integrin) in addition to the EGF receptor and MMP-14 that were expressed in both highly and poorly invasive cells but also, were markedly enriched by HPA pull-down from highly invasive A431 and SW-48 cells, in comparison to poorly invasive HepG2 and RCC4 cells. This finding suggested these proteins were differentially glycosylated with terminal GalNAc between highly and poorly invasive cells.

Interestingly, all the above glycoproteins have been previously implicated to enhance invasion and/or metastasis [53–56]. Moreover, some metastasis suppressor genes (KAI1, also known as CD82) interact with both integrins and the EGFR, and abrogate their signaling, thereby attenuating metastasis [57–59]. Although mechanistic details on how altered glycosylation of these invasion/metastasis promoting glycoproteins enhance cancer cell invasive potential remain unclear, previous studies have shown that glycosylation can modulate the activity of several of these proteins. For example, the EGFR, a transmembrane tyrosine kinase and a therapeutic target, which upon dimerization, activates several signaling cascades including MAPK-, Akt- and JNK-kinase pathways [53]. It also modulates transcriptional program downstream of the EGFR [60]. EGFR is upregulated in many types of cancers [53] and its ectodomain is heavily N-glycosylated [61] and its intracellular domain was shown to be a substrate for O-GlcNAc transferase [62]. Importantly, it is also O-glycosylated by a polypeptide GalNAc-transferases (GALNT2 and GALNT6) that add the first GalNAc to serine or threonine amino acids giving rise to tumor-associated Tn-antigen [63]. Upregulation of GALNT2 and GALNT6 resulted in enhanced migration and invasion of oral and ovarian cancer cells, in part by increasing O-glycosylation and activation of the EGFR [62–64]. Another metastasis-associated glycoprotein whose activity is known to be regulated by altered O-glycosylation is matrix metalloproteinase-14 (MMP-14). Nguyen et al. showed that GALNT1-dependent O-GalNAc glycosylation markedly increased MMP-14 activity, ECM degradation, tumor growth, and invasiveness in a mouse xenograft model [65].

Members of the integrin family of cell adhesion receptors also carry both N- and O-glycans which, when altered, can influence migration and adhesion of tumor cells, a prerequisite for their invasiveness [66–68]. However, direct evidence on the role of O-glycans in integrin-mediated cell adhesion or signaling is almost non-existent. β1 integrin subunit is an exception, and it has been shown to carry core 1 O-glycan(s) a product of the C1GALT1 transferase [63], which is typically overexpressed in hepatocellular carcinomas and HCC cells. Importantly, overexpression of C1GALT1 was found to enhance HCC cell adhesion to ECM proteins, their migration, and invasion, whereas RNAi-mediated C1GALT1 knockdown suppressed this phenotype. By using a mouse xenograft model, the authors also showed that C1GALT1 promotes HCC cell metastasis. These effects were strictly dependent on β1 integrin since the C1GALT1-mediated phenotypic changes were suppressed by the anti-β1-integrin antibody that blocks its activity. An O-GalNAc/GlcNAc modification of αV- and α6 integrin has been reported [51, 52] but their functional significance remains currently unclear.

To summarize, these findings suggest that altered glycosylation of several distinct metastasis-associated glycoproteins, including integrins, EGFR, and MMP-14, is a key to the highly invasive cancer cell phenotype. Yet, further studies are warranted to confirm whether this holds true for all different integrin subunits including those that are overexpressed in cancers but do not display enhanced HPA binding. Our data also highlight the fact that cancer cell invasive potential depends not on a single protein, but rather a compilation of GalNAc-glycosylated proteins, each of them having a special role in invasion, be it cell adhesion, migration, or degradation of the extracellular matrix. Our findings also emphasize the prognostic and therapeutic utility of these invasion-promoting glycoproteins bearing a GalNAc glycotope. However, better identification of the glycotope(s) that bind HPA, and a better understanding of how altered glycosylation regulates the activity of the above metastasis-promoting proteins are also needed before we can rationally address and prevent cancer cell invasiveness in a clinical setting.

## MATERIALS AND METHODS

### Cell culture and sample preparation

All cell lines (COS-7, HepG2, HT-29, SW-48, CaCo-2, DLD-1, MCF-7, MDA-MB 231, RCC4, and A431) were from ATCC (Manassas, VA). Cells were grown in high glucose Dulbecco’s modified Eagle’s medium (DMEM) supplemented with Glutamax (Gibco BRL, Grand Island, NY, USA), 10% fetal bovine serum (HyClone, Cramlington, United Kingdom), and antibiotics (100 U/ml Penicillin and 100 μg/ml Streptomycin: Sigma-Aldrich, St. Louis, MO) in humidified conditions at +37°C and 5% CO_2_.

### Reagents and antibodies

All reagents were purchased from Sigma-Aldrich (St. Louis, MO, USA) unless stated otherwise. The list of antibodies used in this study is present in (Supplementary Table 2).

### Lectin microarray printing and labelling

#### Sample labelling

Cells cultivated on plates to 70–80% confluency were lysed for 30 min on ice with lysis buffer (50 mM sodium tetraborate buffer, pH 8.5, 150 mM NaCl, 1% Triton X-100) supplemented with the protease inhibitor cocktail (Complete Mini, Roche) and clarified by centrifugation (12,000 × *g* at 4°C for 15 min). 12 μg of total protein of each cell lysate was labeled with 6 μg of NHS activated DyLight 633 dye (Thermo Scientific, Waltham, USA) in 50 μl of labeling buffer (50 mM boric acid/150 mM NaCl, pH 8.5) for 1 h at RT with constant agitation (600 rpm). Any extra label was then quenched at RT for 1 h by adding 50 μl of quenching buffer (75 mM ethanolamine in 200 mM Tris–HCl/150 mM NaCl, pH 8.5) before dilution (1:12) with the assay buffer (50 mM Tris/300 mM NaCl/2 mM MgCl_2_/2 mM MnCl_2_/2 mM CaCl_2_, pH 7.1) to give the final pH 7.4 to the labeled sample mix.

#### Staining of microarray slides and quantification

After clearing by centrifugation (12,000 × *g* for 10 min at RT), 400 μl of the labeled sample was applied to each well on a pre-printed (see below) Nexterion H microarray slide (Schott, Germany) embedded in a rubber housing with six separate wells. After further incubation in a humidified chamber with constant agitation for 2 h in RT, wells were washed five times for 5 min each with the washing buffer (50 mM phosphate buffer/0.05% Tween) and finally by dipping the whole slide in washing buffer and water to remove any remaining salt before drying. Array images were generated using the Genepix 4200AL laser scanner (Axon Instruments) with an appropriate filter set for the DyLight 633™ dye. Quantification of the median intensities of the bound label in each spot was done by using the GenePix Pro^®^ microarray analysis software array before calculating the median intensities from three separate wells each having four parallel arrays and three separate lectin spots/array (36 measurement spots/sample). Glycan profiles were then generated by calculating the mean (±SD) from three different samples per cell line.

#### Printing

A Microgrid II array printer equipped with a four pin printer head and in-build software package were used for printing 10 μM lectin stocks (diluted in printing buffer: 50mM phosphate buffer, 1% glycerol and 0, 05% Tween, pH 7.6) on microarray slides. After printing, the slides were allowed to equilibrate for 4 h at RT and 70% humidity before pre-quenching the activated slides with 50 mM ethanolamine and labeling. The lectins (43 different) were purchased from either EY Laboratories (San Mateo, CA, USA) or the Vector Laboratories (Youngstown, OH, USA). All the lectins used are listed in (Supplementary Table 3) with their sugar specificities according to manufacturers’ data sheets. Optimization of the binding specificity and signal-noise ratio was performed using labeled fetuin and asialofetuin as markers. Different lectin-sample ratios were also tested to guarantee unsaturated binding capacity of the lectin spots.

### 3D invasion assay

The invasive properties of each cell type were investigated using an organotypic 3D-myoma-invasion model, as described earlier [41]. In brief, myoma discs pre-equilibrated at +4^o^C in DMEM were placed in tightly fitted Transwell^®^ inserts (Corning, Inc., Corning, NY, USA) after which 5 × 10^5^ cells (in 50 μl of DMEM) were added on top of each disc. After attachment, myoma discs with cells were transferred onto uncoated nylon discs placed on curved steel grids (3 × 12 × 15 mm) in 12-well plates, each well-containing 1ml of DMEM. The myoma organotypic cultures were maintained for 21 days with daily media changes. Each assay was performed in triplicate. The specimens were fixed in 4% formalin overnight, dehydrated, and embedded in paraffin. Finally, 6 μm thick sections were cut and deparaffinized before staining with Mayer’s Hematoxylin-Eosin. After digitalization, the total area and median invasion depth of the invasive foci with cells from each microscopic field were determined by measuring the area and distance of the invasive cell foci from the top cell layer on each disc using the Image J (Fiji) v1.46o software (National Institute of Health, USA).

### Lectin/western blotting

Biotinylated HPA (Sigma-Aldrich) was dissolved in 1× PBS containing 1mM CaCl_2_ and 0.33 mM MgCl_2_ to 1 mg/mL. Cells were lysed in 50 mM Tris-HCl, pH 7.5, 150 mM NaCl, 1% Triton X-100, 1 mM CaCl_2_, 1 mM MgCl_2_ buffer supplemented with the protease inhibitor cocktail (Sigma-Aldrich) on ice for 1 h. Total protein concentration was estimated using BCA Kit (Pierce). Thirty micrograms of total cell lysate protein were resolved in 10% acrylamide gel by SDS-PAGE and then transferred onto the PVDF membrane. To avoid unspecific binding the membrane was incubated in a blocking buffer containing 5% BSA (Sigma-Aldrich) in TBST (TBS with 0.1% Tween-20 (Sigma-Aldrich)) for 3 h at RT. Next, the membrane was probed with 20 μg/mL HPA in 5% BSA, 1 mM CaCl2 in TBST solution for 1h followed by 3 × 10 minutes washing in TBST and incubation with HRP-conjugated streptavidin in 5% BSA, 1 mM CaCl_2_ in TBST solution for 1 h at RT. After washing (5 × 10 minutes each) the positive signal was revealed using SuperSignal™ West Femto Maximum Sensitivity Substrate (Thermo Fisher Scientific).

For the western blotting assay, membranes with transferred proteins were incubated for 1 h in 5% skimmed milk and probed with specific primary antibodies (Supplementary Table 2) overnight at 4°C. Secondary antibodies conjugated with HRP and Lumi-Light Western Blotting Substrate (Roche) were used to visualize specific protein bands. The bands were detected using Fujifilm LAS-3000 bioimaging and scientific research imaging equipment (FUJI Photo film Co., LTD.).

### Pull-down assay

Cells were incubated in lysis buffer (50 mM Tris-HCl, pH 7.5, 150 mM NaCl, 1% Triton X-100, 1 mM CaCl_2_, 1 mM MgCl_2_) supplemented with the protease inhibitor cocktail (Sigma-Aldrich) on ice for 1 h and centrifuged at 17000 × g (4°C) for 15 min. Protein concentration was estimated using BCA Kit (Pierce). To avoid unspecific binding, cell lysates were precleared using Dynabeads™ MyOne™ Streptavidin C1 (Invitrogen). 70 μg of biotinylated HPA was incubated with 0.3 mg of the total cell lysate proteins in 50 mM Tris-HCl, pH 7.5, 150 mM NaCl, 0.1% Triton X-100, 1 mM CaCl_2_, 1 mM MgCl_2_ supplemented with protease inhibitor cocktail for 3 h at 4°C. After this time, 100 μl Dynabeads™ MyOne™ Streptavidin C1 (Invitrogen) was added to the mixture for 1 h at 4°C. Finally, the beads were washed 4 × 1 ml of 50 mM Tris-HCl, pH 7.5, 150 mM NaCl, 0.1% Triton X-100, 1 mM CaCl_2_, 1 mM MgCl_2,_ after which proteins were harvested by adding 2× Laemmli buffer and incubating the beads at 96°C for 5 minutes. Samples were resolved in 10% acrylamide gel followed by silver staining using Silver Staining Kit (Pierce) or probed with a specific antibody in the western blotting assay.

### LC-MS/MS analysis

Silver-stained (Silver Staining Kit (Pierce)) protein bands were cut out of the polyacrylamide gel and digested by adding 0.75 μg trypsin (Sequencing Grade Modified Trypsin, Promega) overnight at 37°C. Cysteine bonds were reduced with 0.045 M dithiothreitol (Sigma-Aldrich) for 20 min at 37°C and alkylated with 0.1 M iodoacetamide (Sigma-Aldrich) at room temperature. Peptides were purified with C18 micro-spin columns (Harvard Apparatus) according to manufactures protocol and dried ones were reconstituted in 40 μl 0.1% trifluoroacetic acid (Sigma-Aldrich) in 1% acetonitrile (Sigma-Aldrich). In the next step, each sample was diluted further 1:4 in 1% acetonitrile, and then 4 μl was injected per LC-MS/MS run. Liquid chromatography coupled with mass spectrometry analysis was carried out on a nanoElute (Bruker Daltonics) coupled online to a hybrid trapped ion mobility spectrometry – quadrupole time of flight mass spectrometer (timsTOF Pro, Bruker Daltonics). Liquid chromatography was performed at 50°C with a constant flow of 400 nL/min using a two-column setup consisting of a 5 mm Acclaim™ PepMap™ 100 C18 trap column (Thermo Fisher Scientific), followed by 25 cm × 75 μm ID, 1.6 μm C18-Aurora emitter column with nanoZero and CaptiveSpray Insert (IonOptics). As the mobile phases, water with 0.1% formic acid (vol/vol; VWR) and acetonitrile with 0.1% formic acid (vol/vol) were applied. Peptides were separated with a linear gradient from 2 to 17% of formic acid within 60 min, followed by an increase to 25% of formic acid within 30 min and further to 37% within 10 min, followed by a 10 min washing step from 37% to 95% and another 10 min wash with 95% formic acid. The timsTOF Pro mass spectrometer was operated in positive PASEF mode using the DDA standard_1.1 sec_cycletime method in acquisition. In brief, MS and MS/MS spectra were recorded from m/z 100 to 1700 and acquired with 100 ms ramp time, 100% duty cycle and 10 PASEF MS/MS scans with precursor target value set to 20 000 a.u. For precursor ions, charge minimum and maximum of 0 and 5 were used, respectively. Range for ion mobility coefficient (1/K0) −0.60–1.60 Vs/cm^2^ was applied. For the number of distinct peptides assigned for each protein by HPA lectin pull-down and LC-MS/MS (Supplementary Table 4).

### Data analysis

Mass spectrometry raw files were processed with FragPipe v15.0 using the protein sequence database of reviewed Human proteins (UniProtKB release 2021_03, Human Proteome UP000005640). Decoy sequences and common contaminants were generated and added to the original database as part of the FragPipe workflow as described in [69, 70]. Trypsin was selected as the cleavage specificity and methionine oxidation and N-terminal acetylation were set as variable modifications. Static residue modification was set for carbamidomethylation of cysteines. The allowed peptide length and mass ranges of 5–50 residues and 200–5000 Da, respectively. Within FragPipe all peptide-spectrum matches (PSMs), peptides, and proteins were filtered to 1% PSM and 1% protein FDR. FDR was calculated based on the hits to decoy database. MSFragger 3.2 precursor and fragment tolerance was set to 20ppm with mass calibration and parameter optimization enabled. Two missed cleavages were allowed, and two enzymatic termini were specified. Isotope error was set to 0/1/2. The minimum number of fragment peaks required to include a PSM in modeling was set to two and the minimum number required to report the match was four. The top 150 most intense peaks and a minimum of 15 fragment peaks required to search a spectrum were used according to recommended settings. Philosopher 3.4.13 was applied for data analysis [71]. Glycosylation status was analyzed using the UNIPROT database and specific for O-glycosylation data collected in [67, 72, 73].

### Statistical analyses

Statistical analysis was performed using either Excel or GraphPad Prism Software (GraphPad Software Inc., La Jolla, CA, USA). Unless stated otherwise, the comparison of medians (± SD) between two groups was done by a two-tailed Student’s *t*-test, whereas multiple groups were compared by one-way ANOVA. All error bars represent standard deviation (SD) and the *p* values <0.05 were considered statistically significant.

### Ethics approval/consent to participate

All uterine leiomyoma tissues were obtained from routine surgeries of otherwise healthy donors after their informed consent. The study protocol was reviewed and approved by the Regional Ethics Committee of the Northern Ostrobothnia Hospital District (license number 2/2017).

### Data availability

The datasets generated during and/or analyzed during the current study are available from the corresponding author (elham.khosrowabadi@oulu.fi) on reasonable request.

## SUPPLEMENTARY MATERIALS




