# Supplementary material for: Altered glycosylation of several metastasis-associated glycoproteins with terminal GalNAc defines the highly invasive cancer cell phenotype
Source: Oncotarget. 2022 Jan 10;13:73–89. doi: 10.18632/oncotarget.28167 (PMC8751650; doi:10.18632/oncotarget.28167)
Supplement: Supplementary file 1 [file oncotarget-13-28167-s001.pdf]

Altered glycosylation of several metastasis-associated glycoproteins with terminal GalNAc defines the highly invasive cancer cell phenotype

SUPPLEMENTARY MATERIALS

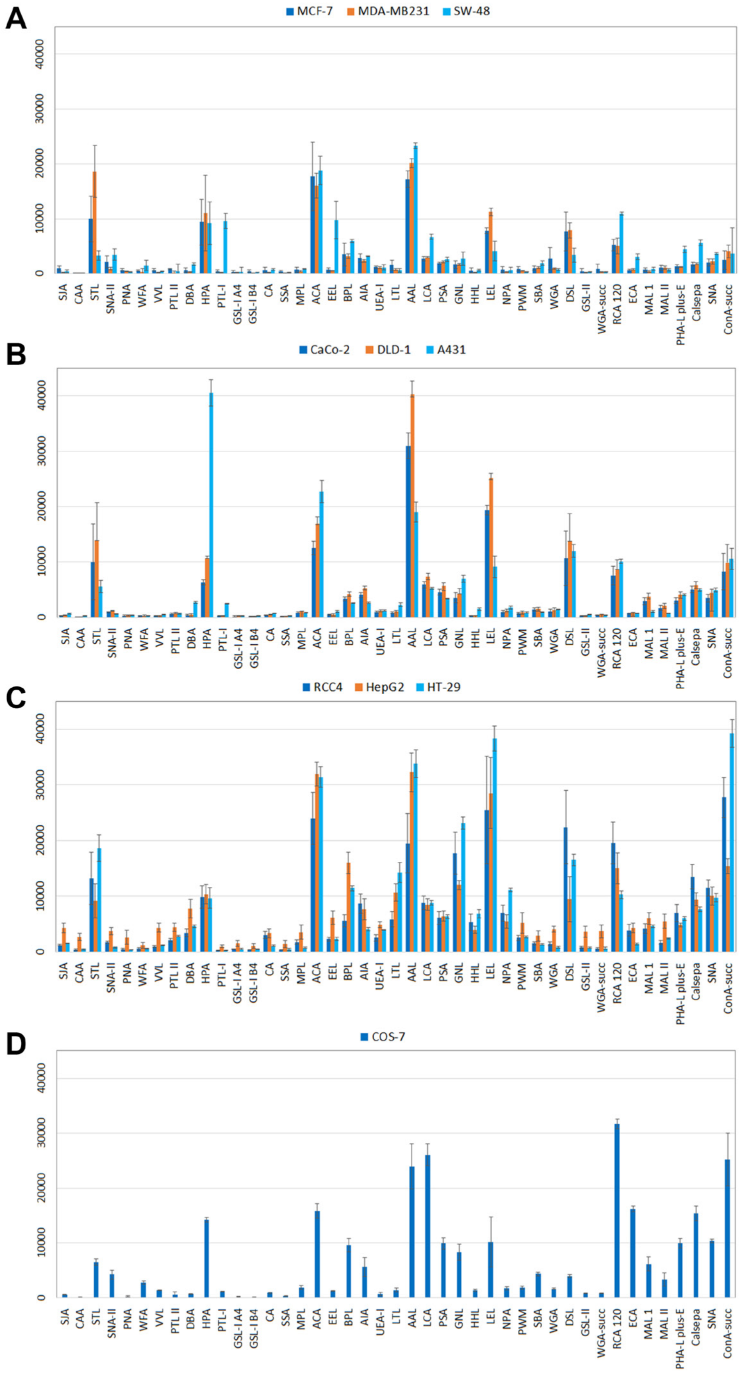

Supplementary Figure 1: Lectin microarray glycan profiles of different cancer cell types. Normalized median binding intensities of three independent samples are shown with standard deviations (SD). (**A–D**) Selected cell lines are grouped in each graph based on hierarchical clustering analyses. Cell lines are denoted at the top of each graph.

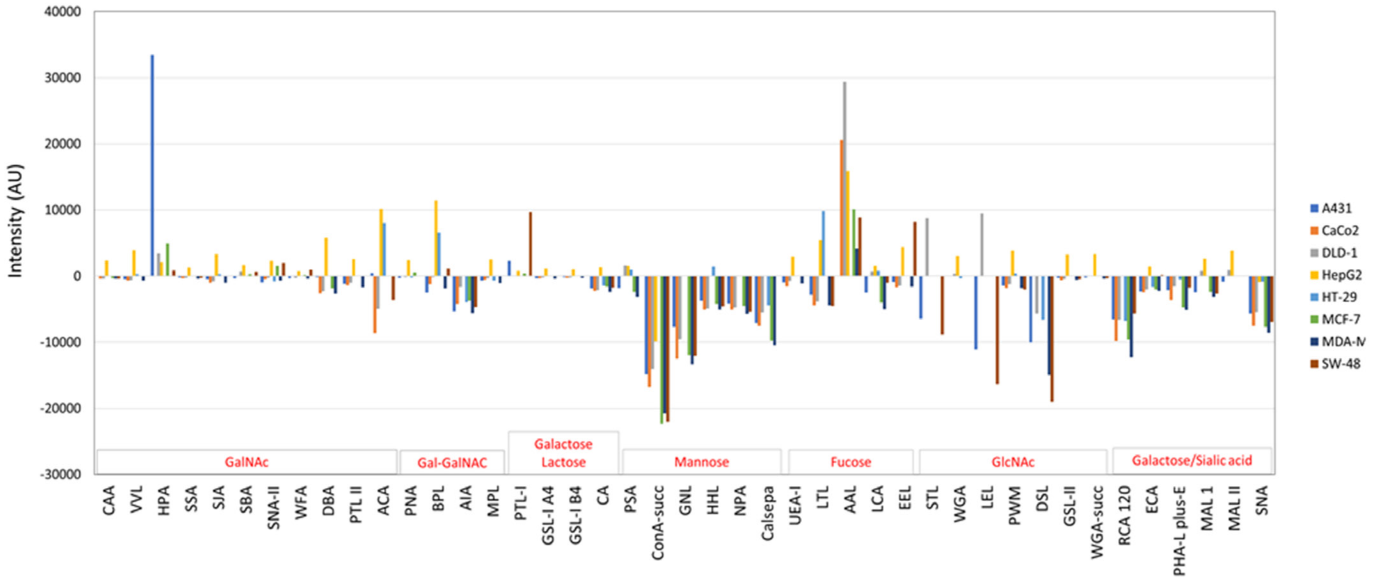

Supplementary Figure 2: Comparison of glycosylation differences in different cancer cell lines in comparison to RCC4 cells. Histograms show differences in lectin binding intensities as subtracted fingerprints between RCC4 cells and the other cancer cell lines. The differences were calculated by subtracting normalized median intensity values of RCC4 cells from those of the other cancer lines. Red boxes denote the main glycotopes recognized by the lectins. Several lectins share the same nominal glycotope binding activity.

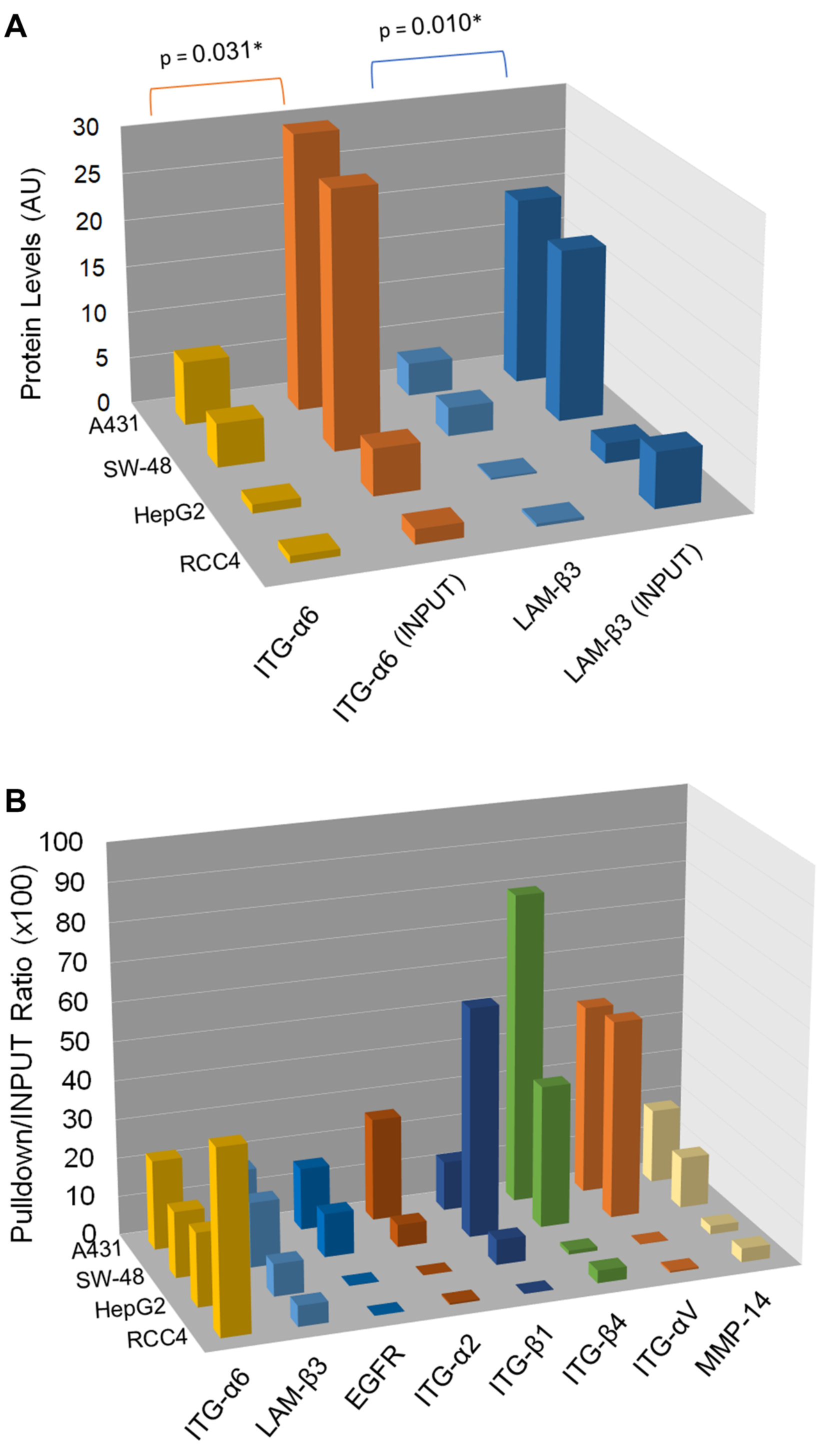

Supplementary Figure 3: Comparison of the HPA pull-down and input protein levels. (**A**) Correlation of α6 integrin and laminin β3 protein levels in HPA pull-down and input protein samples. Correlation and regression analyses showed that in both proteins, protein levels in pull-down and input samples correlate significantly with each other. (**B**) Comparison of pull-down and input protein levels by using their ratios. The graph shows that the ratios between pull-down and input protein levels are much higher in highly invasive cells than in poorly invasive cells. α6 integrin and laminin β3 are the exceptions.

Supplementary Table 1: Cellular proteins identified by HPA lectin pull-down and LC-MS/MS. See Supplementary Table 1

Supplementary Table 2: The list of antibodies used for immunoblotting with their working dilutions and manufacturer's catalog numbers

Name	Company	Cat. No	Dilution
anti-α2 integrin	Santa Cruz Biotechnology	sc-9089	1:1000
anti-α6 integrin	Sigma Aldrich	HPA012696	1:2000
anti-αV integrin	Santa Cruz Biotechnology	sc-6617	1:1000
anti-β1 integrin	gift from Karl Matlin	-	1:1000
anti-β4 integrin	Santa Cruz Biotechnology	sc-9090	1:1000
anti-actin	Sigma Aldrich	A5441	1:4000
anti-EGFR	Cell Signaling Technology	4267	1:1000
anti-laminin β3	Santa Cruz Biotechnology	sc-20775	1:500
Anti-MMP14	Millipore	clone LEM-2/15.8	1:1000

Supplementary Table 3: Lectins and their sugar specificities listed according to manufacturers' data sheets

Lectin	Sugar specificity
CAA	GalNAc
VVL	GalNAc
HPA	GalNAc
SSA	GalNAc (Tn antigen)
GSL-I A4	α-Gal
SJA	GalNAc > Gal
SBA	α/β-GalNAc, Gal
SNA-II	GalNAc = Lac > Gal
WFA	GalNAc >> Lac > Gal
DBA	Terminal α-GalNAc
PTL II	α/β-linked GalNAc, Gal
ACA	GalNAc, Galβ(1,3)GalNAc
PNA	Galβ(1,3)GalNAc
BPL	Galβ(1,3)GalNAc, GalNAc
AIA	Galβ(1,3)GalNAc, α-Gal
MPL	Galβ(1,3)GalNAc > Gal
PTL-I	Gal, α-GalNAc
GSL-I B4	α -Gal
CA	Lac > GalNAc > Gal
EEL	α -Gal(1,3)(α-Fuc(1,2) βGal(1,3/4)βGlcNAc
UEA-I	α-Fuc
LTL	α-Fuc

Lectin	Sugar specificity
PSA	α-Man, α-Glc
ConA-succ	α-Man > α-Glc > α-GlcNAc
GNL	Manα(1,3)Man
HHL	Manα(1,3)Man or Manα(1,6)Man
NPA	α-Man
Calsepa	Man, High Man
AAL	Fucα(1,6)GlcNAc (core Fuc)
LCA	Fucα(1,6)GlcNAc, α-Man
STL	[GlcNAcβ(1,4)]$_{2-4}$
WGA	(GlcNAcβ4)n, NeuAc
LEL	GlcNAcβ(1,4)GlcNAc oligomers
PWM	β(1,4)-linked (GlcNAc)n, N-acetyllactosamine
DSL	(GlcNacβ4)n, tri- and tetraantennary N-glycans
GSL-II	Agalactosylated tri/tetraantennary glycans, GlcNAc
WGA-succ	[GlcNAcβ(1,4)]$_3$ > [GlcNAcβ(1,4)]$_2$ > GlcNAc >> Neu5Ac
RCA 120	Galβ(1-4)GlcNAc-R
ECA	Galβ(1,4)GlcNAC, LacNAc > Lac > GalNAc, Gal
PHA-L plus-E	Gal, Complex triantennary N-linked glycans
MAL-I	(Siaα(2,3)Galβ(1,4)GlcNAc
MAL-II	Siaα(2,3)Galβ(1,4)GlcNAcβMan-R
SNA	Neu5Acα(2,6)Gal or Neu5Acα(2,6)GalNAc

Supplementary Table 4: The number of distinct peptides assigned for each protein by HPA lectin pull-down and LC-MS/MS. See Supplementary Table 4
